# Left ventricle pedunculated thrombi risks and outcomes: a case report and literature review

**DOI:** 10.1590/1677-5449.202301242

**Published:** 2024-08-09

**Authors:** Ahmed Ali Ali, Eman Elsayed Sakr

**Affiliations:** 1 National Heart Institute, Cardiology Department, Giza, Egypt.; 2 Mataria Teaching Hospital, Cardiology Department, Cairo, Egypt.

**Keywords:** left ventricle, myocardial infarction, thrombosis, emboli, risks, outcomes, ventrículo esquerdo, infarto do miocárdio, trombose, embolia, riscos, desfechos

## Abstract

A 42-year-old male with ischemic cardiomyopathy presented with acute bilateral femoral artery embolization. After management with embolectomy and fasciotomy in both femoral arteries, transthoracic echocardiography revealed two pedunculated highly mobile left ventricle (LV) thrombi. Given the procedural risk, anticoagulation therapy was recommended over surgery. However, the bleeding risk impeded the continuation of anticoagulation, which increased the thrombus size. Multiorgan failure and disseminated intravascular coagulopathy followed and the patient died. We also systematically reviewed the PubMed and Scopus databases for pedunculated LV thrombi cases and retrieved 74 and 63 reports respectively. Of these, 37 relevant reports (45 cases) plus 11 reports from the manual search were included for data extraction, a total of 56 cases besides our case. Based on the etiologies and risks, LV thrombi are predictable and preventable, especially after ischemic events. A clear diagnostic algorithm and vigilant follow-up are needed as well as multidisciplinary management once a diagnosis is confirmed.

## Resumo

Relatamos o caso de um homem de 42 anos com cardiomiopatia isquêmica que apresentou embolização aguda bilateral das artérias femorais. Embolectomia das artérias femorais e fasciotomia foram realizadas bilateralmente e, posteriormente, o ecocardiograma transtorácico revelou a presença de dois trombos pedunculados altamente móveis no ventrículo esquerdo (VE). Diante do risco associado à abordagem cirúrgica, recomendou-se terapia anticoagulante. No entanto, o risco de sangramento impediu a continuação da anticoagulação, o que levou ao aumento dos trombos. Posteriormente, o paciente evoluiu com falência de múltiplos órgãos e coagulação intravascular disseminada, vindo a óbito. Além do relato de caso, realizamos buscas sistemáticas nas bases de dados PubMed e Scopus por casos de trombos pedunculados no VE. Foram recuperados 74 e 63 relatos, respectivamente, dos quais 37 relatos relevantes (45 casos) e 11 da busca manual foram selecionados para extração de dados, totalizando 56 casos além do nosso. Com base nas etiologias e nos riscos, os trombos no VE são previsíveis e evitáveis, especialmente após eventos isquêmicos. Isso requer um algoritmo diagnóstico claro e acompanhamento vigilante, bem como manejo multidisciplinar após confirmação do diagnóstico.

## INTRODUCTION

A left ventricle (LV) thrombus is defined as an echo-dense mass near an akinetic or hypokinetic ventricular wall that is visible in at least two different views.^1^ LV thrombi formation following acute myocardial infarction (MI) or dilated cardiomyopathy (DCM) is predisposed by Virchow’s triad (MI-induced endothelial injury and the subsequent elevation of catecholamine levels,^2^ inflammation-triggered hypercoagulability, and blood stasis due to segmental wall motion).^3^ About 6.3% of ST-segment elevation MI (STEMI) cases and 19.2% of anterior STEMI cases with LV ejection fraction (EF) <50% are complicated with LV thrombus formation within two weeks to three months of the onset of myocardial injury. Other risk factors of LV thrombus formation are dilated heart failure, hypercoagulable states, nonischemic cardiomyopathy, and Takotsubo cardiomyopathy.^4^ LV thrombi develop in 1.3%^5^ to 2.2%^4^ of patients with acute Takotsubo cardiomyopathy and are significantly associated with the presence of both apical ballooning and high troponin level >10 ng/mL. Pedunculated LV thrombi have a higher embolic potential than mural thrombi, depending on the extent of protrusion into the left ventricle, mobility, and the pedunculated shape. There is no theory explaining the exact mechanism or combination of factors that favor the formation of pedunculated thrombi. Nonetheless, the literature has reported many mural thrombi that partially detached and transformed into pedunculated thrombi during follow-up or hospitalization.^6-8^ Herein, we depict a case of pedunculated LV thrombus that presented with bilateral acute lower limb ischemia and review the literature for similar case presentations highlighting the main risks and outcomes.

## METHODOLOGY

We searched PubMed and Scopus databases using the keyword search terms “left ventricle OR left ventricular” AND pedunculated AND “thrombus OR thrombi”. All published reports presenting cases of pedunculated left ventricular thrombus were included with no restriction on age or year of publication.

## RESULTS

### Presentation of case

A man in his 40s with a history of diabetes mellitus, systemic arterial hypertension (SAH), and ischemic heart disease with resultant ischemic heart failure presented with bilateral lower limb pain and loss of motor and sensory activity. The patient was evaluated in the emergency department and the evaluation revealed acute bilateral lower limb ischemia that prompted an immediate surgical intervention.

**Figure 1:**
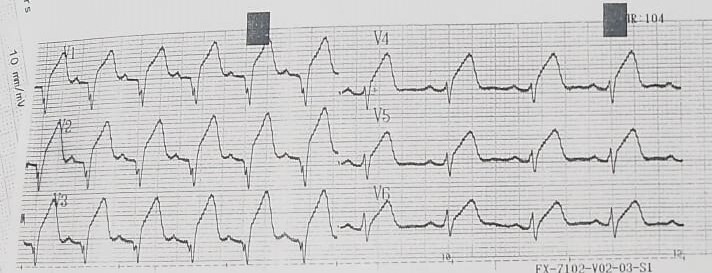
Electrocardiogram of the patient five years before his recent presentation showing ST-segment elevation myocardial infarction in leads V1-6 after receiving thrombolysis (streptokinase), which failed.

Five years before the current presentation, the patient reportedly had extensive anterior STEMI (Figure 1) that was treated with streptokinase and rescue percutaneous intervention (PCI). Coronary angiography showed 80% stenosis of the proximal left anterior descending (LAD) artery, for which a drug-eluting stent was deployed. The patient’s transthoracic echocardiography (TTE) showed an EF of 45% with an akinetic apical and mid septum, apical and mid anterior, and apical inferior segments; normal LV dimensions; and dilated left atrium. Over four years, the patient had deteriorating heart failure with EF 30%, akinetic anteroseptal and mid to apical septal segments, a restrictive pattern of diastolic function, dilated LV and left atrium, and mild mitral regurgitation.

On examination, the vital parameters recorded systolic blood pressure of 90 mmHg, heart rate of 89/min, respiratory rate of 18/min, and temperature of 36. Laboratory investigations were notable for leukocytosis (15,300 cells/microliter), elevated cardiac enzymes (CK-MB 130 U/L; troponin I 71, 6-fold the upper normal limit), and an international normalized ratio (INR) of 1. Other laboratory test results were normal (hemoglobin, platelets, serum creatinine, serum urea, arterial blood gas). Arterial duplex revealed bilaterally damped monophasic flow across the external iliac artery down to the superficial femoral artery. Furthermore, there was no detectable flow distally down to the infra-popliteal arteries. The patient’s acute limb ischemia was managed surgically by bilateral mechanical thrombectomy and fasciotomy.

**Figure 2:**
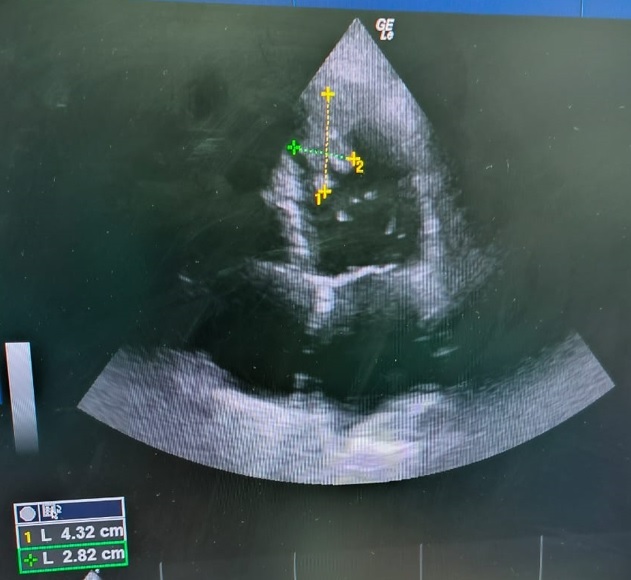
Transthoracic echocardiography; a four-chamber view of the heart after the bilateral mechanical thrombectomy and fasciotomy showing a pedunculated mass (thrombus) measuring 4.32*2.82 cm.

A TTE was obtained after the operation and elucidated decreased EF (20%, measured by M-mode) with global hypokinesia and a large pedunculated irregular hypermobile LV thrombus at the LV apex measuring 4.32*2.82 cm (Figure 2). An electrocardiogram revealed left bundle branch block and prolonged QT interval.

**Figure 3:**
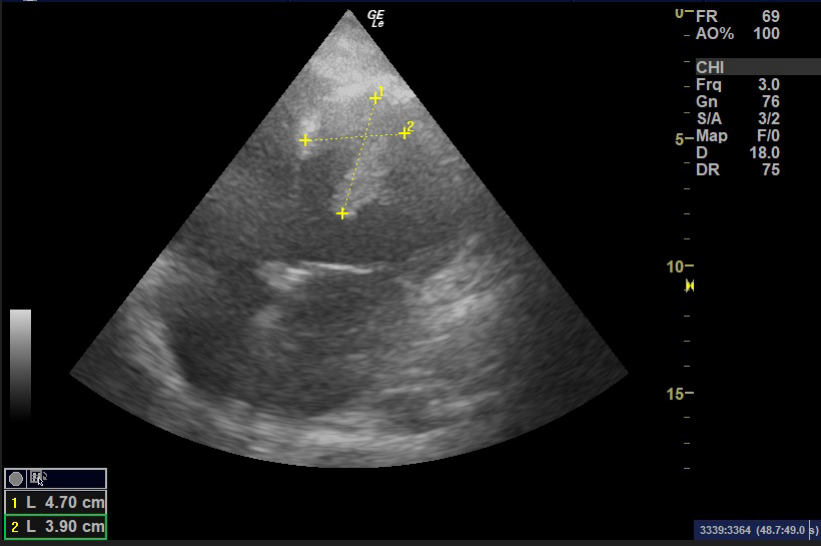
A four-chamber view of the patient’s heart a few days after admission showing enlarging thrombus size (4.7*3.9 cm).

Cardiothoracic surgery consultation refused the surgery for the procedural risks and, because of this, anticoagulation treatment (with warfarin 5 mg once daily and enoxaparin sodium 100 mg twice daily) was decided on after counseling with the patient. Soon after the operation, the patient’s condition deteriorated and required endotracheal intubation. The deterioration entailed circulatory collapse and multiorgan failure (acute kidney injury, ischemic/shocked liver, and disturbed consciousness level). Furthermore, the patient exhibited a bleeding tendency (hematemesis, melena, and nasal bleeding), which necessitated discontinuation of the anticoagulation. As a consequence, the thrombus increased in size to measure 4.7*3.9 cm (Figure 3) by TTE. The laboratory test results were then notable for low hemoglobin (7.4 g/dl), low platelets (87* 10^3^/UL), elevated renal function test values (serum creatinine 6 mg/dl; urea 267 mg/dl); elevated liver function test values (AST 1145; ALT 1063), and elevated serum potassium (6.5 mmol/dl). The patient eventually died. This manuscript conforms to the Helsinki Declaration and local ethical guidelines.

### Systematic review of literature

**Figure 4:**
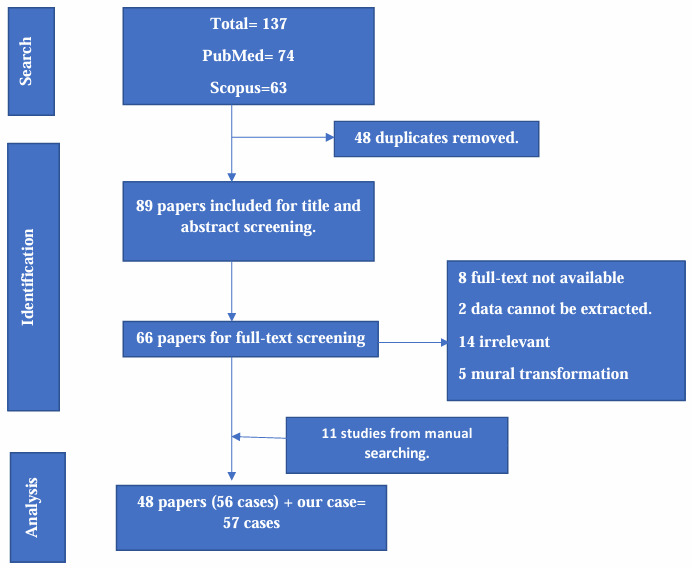
Flowchart of selection and screening of relevant studies.

Searches of PubMed and Scopus databases retrieved 74 and 63 articles, respectively. After removing duplicates, 90 records were eligible for title and abstract screening, which revealed 66 reports to be eligible for full-text screening. Only cases reporting pedunculated thrombus/thrombi at the first assessment for any etiology were included; records with irretrievable data were excluded; reports of mural thrombus/thrombi that transformed into pedunculated thrombi during follow-up were also excluded for carrying different risks. Only 45 cases from 37 papers met our inclusion and exclusion criteria. A manual search revealed 11 more pertinent cases. Our report was included in the analysis (Figure 4)/ flowchart), reaching a final total of 57 cases analyzed.

Baseline demographic and clinical data, management, and outcome are summarized in the Appendix, Table 1. Overall, the mean age of all the cases was 50.5±15.6 years, and 66.6% (38/57) were male. Based on etiology, 41.1% of cases (N=24) were attributed to old (N=12) or acute (N=12) MI; 17.5% (N=10) to cardiomyopathy (non-ischemic DCM, Takotsubo cardiomyopathy, peripartum, or idiopathic cardiomyopathy); and 7% (N=4) were due to heart failure. Other etiologies were ulcerative colitis (N=2), COVID-19 infection (N=2), and coagulopathy (N=6). About 17.5% (N=10) developed idiopathic LV thrombi. Combined etiologies were also found.^59,60^

Twelve (21%) of the cases had a previous coronary artery disease, 11 (19.2%) had SAH, seven (12.2%) had a smoking history, and three (5.1%) had a history of substance abuse (alcohol, anabolic androgenic steroids, and cocaine). Furthermore, seven (12.2%) had diabetes mellitus, five (8.7%) had coagulopathy (essential thrombocytopenia, polycythemia vera, and cystic fibrosis for example), and four (7%) had hyperlipidemia. Of note, seven cases (12.2%) had no identifiable risk factors.

The vast majority of LV thrombi were located at the apex (73.6%; 42/57) followed by the interventricular septum (8.7%; 5/57). However, some cases exhibited more than one thrombus at two or more sites and three cases did not report the thrombus site (Table 2). About 16 (28%) cases developed LV thrombus despite having normal systolic function on presentation. The etiology of the LV thrombus was idiopathic in 10/16 of these cases, due to a hypercoagulable state in two cases, and due to ulcerative colitis inflammatory condition in two cases. It is worth stating that SAH was a risk factor in 4/16 (25%), and the ECG showed abnormal changes in only 3/16 (18.7%) of these cases.

Thirty-three patients (57.8%) developed distal emboli at different sites, the most common of which was the brain (N=14), followed by the arterial system of the lower limb (N=13). Other sites are listed in Table 1 (see the Appendix). The definitive treatment for most cases was surgical removal (75.4%; N=43). Furthermore, three cases responded well to oral anticoagulation with warfarin. While five cases responded well to heparin infusion, four did not show any improvement, and the management plan was changed to surgery, tirofiban, recombinant tissue plasminogen activator (RTPA), or streptokinase.

## DISCUSSION

Despite the absence of histopathological confirmation, our patient was diagnosed with LV thrombus because of his associated factors (history of anterior STEMI with reduced EF) and current presentation. Surgical removal is the definitive management for mobile pedunculated masses because of their high embolization risk.^61^ Alternatively, for cases that refused surgery or were deemed to be unfit for surgery, direct oral anticoagulants were non-inferior to vitamin K antagonist (warfarin) for treating LV thrombus.^61^ Based on our review, surgical removal was almost always successful, whereas oral and intravenous anticoagulation was relatively less successful (5/12; 41.6% failure rate). Furthermore, the three cases that died, including our case, were managed using anticoagulation and did not proceed to surgery.

There are no clinical trials assessing the efficacy of thrombolysis via streptokinase, RTPA, or urokinase. Nonetheless, thrombolysis carries a high embolic and hemorrhagic risk despite the potential for successfully dissolving the LV thrombus.^62^ The deterioration of the LVEF seen in our patient can be explained by the thrombus, given that the LVEF improves or even normalizes after LV thrombus removal or dissolution in some other patients.^63-70^ This finding has been confirmed earlier where the LAD/anterior wall infarctions were significantly associated with contractile dysfunction at the apex and with decrements in the peak systolic function.^71^

Four-dimensional magnetic resonance imaging (4D MRI) for intracardiac hemodynamic assessment was tested in anterior MI cases, which is the most reported risk for LV thrombi. It revealed reduced peak systolic flow in the mid ventricle and apex and reduced peak diastolic flow in the apex in anterior acute MI.^72^ This explains the occurrence of apical thrombi in anterior/LAD MI. However, there is no reported prophylactic anticoagulation strategy to date. Accordingly, any use of prophylactic anticoagulation should be tailored on a patient-by-patient basis. Notably, prophylactic anticoagulation in LV thrombus was a class IIb recommendation according to the 2013 ACC/AHA STEMI guidelines.^73^ Low-dose anticoagulation with rivaroxaban 2.5 mg BID for 30 days, besides dual antiplatelet therapy (DAPT), has been tested recently.^74^ The low-dose rivaroxaban plus DAPT cohort had a lower incidence of LV thrombus formation than the DAPT alone cohort (0.7% and 8.6%, respectively; hazard ratio 0.08). On the other hand, anticoagulation has no role in preventing LV thrombus formation in DCM with sinus rhythm.^75^ The Heart Failure Long-Term Antithrombotic Study (HELAS) trial has also compared the incidence of thromboembolism with warfarin, aspirin®, or placebo in chronic heart failure.^76^ There was no significant difference in the incidence between the three groups.

TTE was the major diagnostic tool used in the literature cases and also in our case. Noting the difficulty of diagnosing mural thrombi and their potential of transformation into pedicled thrombi, routine assessment of patients following MI (particularly anterior MI with reduced LVEF) or cardiomyopathy diagnosis is advised for early diagnosis and management. Delayed enhancement cardiac magnetic resonance imaging (DE-CMRI) was revealed to be the most sensitive imaging modality for detecting LV thrombi and distinguishing them from the normal myocardium.^77^ It had a significantly higher performance than both the standard TTE and cine-CMRI for detecting LV thrombi.^78^ DE-CMRI has a 100% negative predictive value and 100% sensitivity.^79^ The absence of vascularity in the thrombi prevents late gadolinium enhancement on CMRI from increasing the sensitivity and specificity of the modality. Nonetheless, DE-CMRI cannot be afforded for all acute MI patients. So, an algorithm entailing routine non-contrast echocardiography for stratifying patients based on apical wall motion score was proposed; the presence of apical wall motion then warrants performing DE-CMRI.

Strengths and limitations: the strengths of this article lie in the complicated presentation and review of all the previously reported cases of pedunculated LV thrombi. The evidence level of each of the records from which data were extracted is given in the Appendix, Table 1, according to the Oxford Center for Evidence-based Medicine.^80^ In our case, the main limitation was the delayed access to TTE, which was only performed after the operation. The delayed diagnosis of the LV thrombus prevented early multidisciplinary management and early discussion between the vascular and cardiothoracic teams to decide on the optimum management plan.

## CONCLUSION

This report highlights a pedunculated LV thrombus in a case of ischemic cardiomyopathy with a previous history of acute anterior MI. Multidisciplinary management is a cornerstone in managing similar complicated cases. Early surgical management of pedunculated LV thrombi is the management of choice and it should be considered to avoid the failure rates of anticoagulation and thrombolytic medications. A clear diagnostic algorithm should be adopted for early diagnosis and for avoiding embolic presentations. Similarly, screening algorithms should also be developed for patients with non-ischemic cardiomyopathies and those liable to LV thrombosis with normal LV function - inflammatory bowel disease, and hypercoagulable states, for example. Furthermore, large clinical trials of the efficacy of prophylactic anticoagulation following acute MI, specifically anterior/LAD MI, are needed.

## Appendix

Data on baseline characteristics, presentation, ECG, possible cause, management, and outcome. (ECG, electrocardiogram; TIA, transient ischemic attack; MI, myocardial infarction; STEMI, ST-segment elevation myocardial infarction; MCA, middle cerebral artery; NR, not reported; HF, heart failure; LVAD, left ventricle assist device; AKI, acute kidney injury; BiVAD, biventricular assist device; IHD, ischemic heart disease; PCI, percutaneous intervention; LAD, left anterior descending artery; FUO, fever of unknown origin; LCX, left circumflex artery; LV, left ventricle; CABG, coronary artery bypass graft; NYHA, New York heart association; NSTEMI, non-ST-segment elevation myocardial infarction; CoVID-19, coronavirus disease 2019; IV, intravenous; RTPA, recombinant tissue plasminogen activator; AF, atrial fibrillation; DVT, deep vein thrombosis; RVOT, right ventricle outflow tract; DCL, disturbed consciousness level; RBBB, right bundle branch block.

**Table 1:** Data on baseline characteristics, presentation, ECG, possible cause, management, and outcome. (ECG, electrocardiogram; TIA, transient ischemic attack; MI, myocardial infarction; STEMI, ST-segment elevation myocardial infarction; MCA, middle cerebral artery; NR, not reported; HF, heart failure; LVAD, left ventricle assist device; AKI, acute kidney injury; BiVAD, biventricular assist device; IHD, ischemic heart disease; PCI, percutaneous intervention; LAD, left anterior descending artery; FUO, fever of unknown origin; LCX, left circumflex artery; LV, left ventricle; CABG, coronary artery bypass graft; NYHA, New York heart association; NSTEMI, non-ST-segment elevation myocardial infarction; CoVID-19, coronavirus disease 2019; IV, intravenous; RTPA, recombinant tissue plasminogen activator; AF, atrial fibrillation; DVT, deep vein thrombosis; RVOT, right ventricle outflow tract; DCL, disturbed consciousness level; RBBB, right bundle branch block.

**Author/ year**	**Age (year)**	**Gender**	**Risk factors**	**Presentation**	**ECG**	**Extracardiac emboli site**	**Cause**	**Management**	**Outcome/ complications**	**Evidence level**
Kumar et al., 2016^9^	44	Male	Smoking	TIA (upper limb weakness)	old anterolateral MI (t wave inversion and downward ST sloping v1-4)	brain	Old anterolateral STEMI	surgical removal	Recovery and discharge.	4
Grewal et al., 2020^10^	23	Female	ulcerative colitis (diagnosed 2-3 weeks before presentation)	Stroke (sudden weakness on the right side with aphasia; left MCA infarction) followed by episodes of TIA despite anticoagulation therapy	Normal	brain, MCA	Ulcerative colitis	Surgical removal	Recovery and discharge.	4
Garg et al., 2021^11^	60	Male	Hypertension, schizophrenia	fever, dyspnea, desaturation (83% room air), altered consciousness	Sinus rhythm, left ventricular hypertrophy, prolonged QTc	mural thrombus and pulmonary embolism	CoVID-19 pneumonia	Heparin	NR	4
Cousin et al., 2014^12^	63	Male	HF	cardiogenic shock and hypotension	NR	None	Non-ischemic dilated cardiomyopathy	Surgical thrombectomy and LVAD	Recovery and discharge (with a plan for heart transplantation)	4
	50	Male	Biventricular HF and coagulopathy	dyspnea, bilateral lower limb edema, pneumonia, septic shock, and AKI	NR	None	HF/ coagulopathy (occluded right popliteal vein)	Surgical thrombectomy and BiVAD. Heart transplantation after 6 months.	Recovery and discharge	4
	64	Male	IHD, HF, chronic lymphocytic leukemia, and acute promyelocytic leukemia	NR	NR		IHD and HF	Surgical removal	Thrombus recurrence after 6 months of recovery.	4
Kanazawa et al., 2016^13^	75	Female	NR	NR (referral)	Q waves v1-v4	None	Apical aneurysm due to asymptomatic MI	Surgical removal	NR	4
Allende et al., 2011^14^	74	Female	essential thrombocythemia and previous unstable angina (PCI to the LAD)	Atypical chest pain and left hemiparesis (on the 2nd day of admission)	Normal then negative T wave	Brain and heart (distal LAD)	Combined essential thrombocythemia with IHD.	Surgical removal and saphenous vein graft to the distal LAD.	Improvement (of neurological symptoms) and discharge	4
Lutz et al., 2007^15^	34	Male	hyperlipidemia, gastroesophageal reflux disease, pyelonephritis, hydronephrosis, Crohn’s disease, ischemic colitis, and depression	Referral, FUO	NR	None	Ulcerative colitis	Surgical removal	Recovery and discharge (4 days)	4
Nili et al., 1988^16^	59	Male	Eight-month history of stable angina	Chest pain, acute anteroseptal MI followed by HF	NR	None	Acute MI and HF	Surgical removal and graft of the LAD	Recovery and discharge (14 days)	4
	56	Male	Polycythemia vera	left upper quadrant abdominal pain and fever for two weeks after anterior MI (treated by heparin infusion).	NR	Spleen	Acute MI and HF	Surgical removal of thrombus and double coronary bypass (LAD and LCX)	Recovery and discharge (22 days)	4
	46	Male	MI 1 year before presentation	Left common femoral artery occlusion	NR	Common femoral artery	MI (1 year before presentation)	Surgical removal of LV thrombi (thrombectomy)	Recovery and discharge (10 days)	4
	66	Male	eleven-year history of angina; MI 2 years; CABG candidate	Angina	NR	None	MI (2 years)	Surgical removal then CABG	Recovery and discharge (12 days)	4
Kharwar et al., 2014^17^	30	Female	Pregnancy (hypercoagulable state) with poor LV function	peripartum cardiomyopathy Orthopnea and dyspnea on exertion (3 weeks after delivery)	Sinus tachycardia	None	Peripartum cardiomyopathy (poor LV function and hypercoagulable state)	Oral anticoagulation (warfarin)	Complete dissolution (30 days) and improvement of systolic function to 43%	4
Ito et al., 2022^18^	52	Female	IHD	Discovered during an MRI study	NR	None	MI (15 years)	Surgical removal	recovery and discharge (10 days)	4
Singal et al., 2021^19^	32	Male	Two-year history of anabolic androgenic steroid abuse and three-month history of mephentermine abuse.	Acute decompensated heart failure (plus left upper limb monoparesis and embolic TIA on the second day)	Sinus tachycardia and left ventricle enlargement	Brain (left parietal lobe and right cerebellum)	Toxic cardiomyopathy (secondary to mephentermine and/or anabolic androgenic steroid abuse)	Anticoagulant (warfarin)	Complete dissolution (with an improvement of NYHA classification and LV function after two weeks)	
Tanaka et al., 2014^20^	37	Female	Pregnancy (hypercoagulable state) with poor LV function	Exertional dyspnea and fatigue	NR	None	Peripartum cardiomyopathy (poor LV function and hypercoagulable state)	Surgical removal	Recovery and discharge (day 10)	4
Jeganathan et al., 2011^21^	62	Male	Hypertension, renal impairment, and colon cancer treated surgically (4 years)	Right acute limb/leg ischemia with compartment syndrome	NR	Right popliteal artery	Idiopathic	Surgical removal	Recovery and discharge	4
Janula et al., 2021^22^	47	Male	Diabetes mellitus, obesity, and dyslipidemia	NSTEMI in the context of CoVID-19 infection, fever, and expressive aphasia developed during hospital stay (day 4)	RBBB	Right occipital and left temporal regions (with micro-hemorrhagic transformation, contraindication for anticoagulation)	procoagulant state of CoVID-19 and acute MI	Surgical removal	Recovery	4
Marchini et al., 2009^23^	33	Female	Hypertension, smoking, and repeated miscarriage	Dyspnea and lower limb edema for 3 years (NYHA II)	Q waves I, aVL; st depression II and III; T wave inversion I, aVL, V5, and V6; and LV hypertrophy	None	HF	Surgical removal	Recovery (discharge 9 days)	4
Mukai et al., 1991^24^	68	Male	Diabetes	Congestive heart failure (thrombus developed during the hospital stay, on the 15^th^ day)	Sinus tachycardia, mild LV hypertrophy	None	Dilated cardiomyopathy	Surgical removal	Recovery	4
Park et al., 1986^25^	33	Male	IHD (inferior STEMI 6 years before presentation), emboli to the right femoral artery and left internal iliac artery (failed bypass and right above-knee amputation)	Mesenteric artery thromboembolism (abdominal pain)		mesenteric artery	MI (6 years)	Surgical removal	Recovery without recurrence (complicated with an embolus to the left femoral artery on the 20^th^ day, which was treated with left above-knee amputation.	4
Bakhtiari et al., 2012^26^	51	Male	Diabetes mellitus, hypertension, hyperlipidemia, IHD (MI 3 years prior)	Two-week history of right-sided weakness, left-sided paresthesia, and visual disturbance bilaterally.		Brain (bioccipital, basal ganglia, and internal capsule)	MI (3 years)	Surgical removal	Recovery	4
Chen et al., 1981^27^	74	Male	IHD, ventricular ectopy, congestive cardiomyopathy, and diabetes mellitus	Dysarthria, weakness, and ataxia		Brain	Idiopathic congestive cardiomyopathy	IV heparin	Death	4
Rester et al., 2001^28^	23	Female	Pregnancy-induced hypertension and peripartum cardiomyopathy	Fatigue, shortness of breath, and bilateral flank pain.	Sinus tachycardia with non-specific ST-T segment abnormality	Spleen, right kidney	Peripartum cardiomyopathy (poor LV function and hypercoagulable state)	RTPA (after failure of heparin infusion and enlargement of the thrombus size)	Recovery (complete lysis of the thrombus after 8-10 hours)	4
Azari et al., 2021^29^	45	Male	Diabetes mellitus, hypertension, smoking, and alcohol intake	Negligible MI (severe epigastric pain, fever, sweating, and tachycardia)	Q waves in V1-2 and biphasic T wave V2-4	mesenteric artery	Acute MI and HF	Surgical removal	Recovery	4
Chen et al., 2008^30^	84	Male	IHD (anterior MI 8 years ago), and AF	Acute left lower limb ischemia	NR	Left lower limb	MI (8 years)	Surgical removal	NR	4
Kumar et al., 2022^31^	57	Male	None	Bilateral acute lower limb ischemia with absent dorsalis pedis and posterior tibial artery pulses bilaterally	Normal	mid and distal anterior tibial and dorsalis pedis bilaterally	Idiopathic	Aspirin® (150 mg), clopidogrel (75 mg), and LMWH for 48 hours/ till leg numbness disappeared. Then antiplatelet plus dabigatran 110 mg for two weeks (till the thrombus disappeared). Resumed on aspirin® plus dabigatran 150 mg twice daily for 6 months.	Recovery of leg condition and lysis of the LV thrombus	4
Eren et al., 2013^32^	45	Female	NR	Cerebrovascular accident (loss of consciousness for 15 minutes followed by ataxia)	Normal (sinus rhythm)	Brain (bilateral infarcts)	Idiopathic	Surgical removal	NR	4
Daley et al., 1987^33^	40	Male	Agnogenic myeloid metaplasia	Maculopapular rash, fever, and pleuro-pericardial pain	Normal	None	Idiopathic myocarditis and spontaneous platelet aggregation.	Surgical removal	Recovery and discharge (4 weeks)	
John et al., 1991^34^	63	Female	Peptic ulcer	MI	Inverted T wave I, aVL, and V2-6	None	Acute MI	Surgical removal	Recovery and discharge.	4
	56	Male	NR	Unstable angina (4 weeks)	Q waves anteriorly	None	Acute MI	Surgical removal	Recovery and discharge.	4
Lewin et al., 1980^35^	51	Male	IHD (inferior MI 6 years and anterolateral MI 4.5 years backward)	Bilateral acute limb ischemia	Old inferior and anterior MI (persistent ST elevation)	bilateral to the Iliac arteries	Aneurysm of the anterior wall	Surgical removal of the thrombus with aneurysmectomy	Recovery and discharge (14 days)	4
Shetty et al., 2011^36^	49	Female	Anxiety, hypertension, and surgical history of hysterectomy.	One month history of exertional dyspnea	NR	None	prothrombin G20210 mutation	Surgical removal followed by anticoagulation (enoxaparin and warfarin then warfarin).	Recovery and discharge. Complicated by atrial mass after 6 weeks, dissolved medically after two months of medical treatment	4
Vaganos et al., 1989^37^	43	Female	history of bilateral DVT and pulmonary emboli.	Eight-hour history of pain, pallor, pulselessness, and paralysis of the left leg.	Normal	Left common femoral artery	Possible hypercoagulable state	Surgical removal	Recovery	4
Lew et al., 1983^38^	63	Male	history of DVT and pulmonary embolism ( the patient was already on IV heparin)	Acute right limb ischemia		Right femoral artery	Possible hypercoagulable state	Surgical removal	Recovery	4
Chamsi-Pasha et al., 2009^39^	32	Male	None	Routine echocardiography (asymptomatic)	Normal	None	Idiopathic cardiomyopathy	Warfarin overlapped with enoxaparin maintaining INR 2-3, then warfarin for 6 months	Complete dissolution after 6 weeks	4
Early et al., 2001^40^	64	Female	NR	Acute anterior MI, received thrombolytic therapy + no LV thrombus on presentation	Anterior STEMI	None	Acute MI	Surgical removal	Recovery	4
Wohlfarter et al., 1991^41^	28	Male	Appendectomy (4 weeks)	Occlusion of the left superior femoral A at the adductor canal (dragging pain at the cuff)		Left superior femoral artery		Heparin failed, systemic thrombolysis with streptokinase 750000 IU replaced by ancrod 70 IU/day IV for increased movement, then heparin 3000 IU IV	Recovery (thrombus size decreased to 0.7 after 5 days, then it disappeared after two weeks)	4
Palazzuoli et al., 1994^42^	70	Male	bilateral lower limb arteriopathy.	Episodes of disorientation	Recent inferior MI	None	Acute MI	Calcium heparin 12500 IU/ 8 hours	Complete dissolution after 20 days.	4
Jeon et al., 2012^43^	40	Male	None	Stroke and acute limb ischemia (dyspnea, right facial and limb weakness as well as both lower limb pain, pulselessness and coldness).	Sinus rhythm with diffuse non-specific ST segment changes.	Brain, right femoral and right popliteal, and left popliteal arteries	Idiopathic dilated cardiomyopathy	Surgical removal	Recovery and discharge (7 days)	4
Chirillo et al., 1996^44^	47	Male	Recurrent pulmonary embolism, DVT (bilateral femoral and saphenous), smoking, and left lung cancer	Bilateral pulmonary embolism, left pulmonary infarction masking tumor, and thrombosis in the IVC (Sudden dyspnea, tachycardia, and hypoxemia)	RBBB	Lung, lower limb, IVC	Hypercoagulable state resistant to anticoagulation (paraneoplastic hypercoagulable state)	Heparin and RTPA after 5 days	Death (electromechanical dissociation due to RVOT obstruction)	4
DeWitt et al., 1988^45^	80	Female	None	Stroke (right upper limb weakness and speech abnormality)	Nonspecific ST and T wave changes	Brain	Idiopathic	Heparin then warfarin.	Recovery and dissolution of thrombus (12 days)	4
Hwang et al., 1985^46^	43	Male	None	Anterior MI (2 weeks)	NR	Superior mesenteric artery	Acute MI	Surgical removal	Recovery and discharge.	4
Çil et al., 2013^47^	28	Male	IHD (anterior MI 2 years)	Deteriorating dyspnea (decompensated heart failure NYHA class IV)	Anterior ST-segment elevation	None	Essential thrombocythemia, previous IHD with resultant aneurysm, and HF	Tirofiban after failed heparin infusion (1000 IU/hour for 48 hours)	Complete dissolution after 48 hours (decreased size after 24 hours to 1.8*0.7)/ recovery and discharge (on the 6th day)	4
Seitz et al., 2012^48^	48	Male	Cystic fibrosis	DCL, hemoptysis, dyspnea, and respiratory arrest	NR	None	Takotsubo cardiomyopathy	Surgical removal	Recovery and discharge (on day 5 after the operation)	4
Manasrah et al., 2022^49^	54	Female	Type II DM and smoking	Two-hour history of right leg pain.	RBBB	The aortoiliac bifurcation, bilateral common iliac arteries, and proximal left internal iliac artery	Idiopathic	Surgical removal	Recovery and discharge	4
Jeganathan and Ralph‐Edwards, 2011^50^	62	Male	Hypertension, colorectal carcinoma, and renal impairment.	Acute right leg ischemia	NR	Right popliteal artery	Idiopathic	Surgical removal (after heparin failed)	Recovery and discharge	4
Erkal et al., 2017^51^	63	Male	Right femoral embolectomy 2 weeks before presentation	Left femoral artery occlusion	Normal sinus rhythm	Left femoral artery	Idiopathic	Medical treatment	Recovery	4
Maruri-Sánchez et al., 2019^52^	38	Male	Smoking, dyslipidemia, hypertension, and left lower limb DVT 2 years ago	Stroke	NR	right MCA	Idiopathic	Surgical removal	Recovery and discharge	4
Muller et al., 1996^53^	41	Female	Hypertension	Stroke	Left anterior hemiblock, and non-specific ST-T wave changes	Left MCA	Idiopathic	Surgical removal	Recovery (the patient had post-operative wound infection)	4
Lin et al., 2004^54^	23	Male	None	MI (acute proximal LAD lesion), TIA, transient loss of vision 5 min the day before presentation.	Q waves V1 to V5 and low voltage limb leads	Brain (TIA)	Acute MI and premature coronary artery disease	Heparin then surgical removal	Recovery and discharge	4
Kuroki and Murakami, 2012^55^	58	Female	None	MI (acute LAD occlusion), chest pain for two days	Anterior ST elevation	None	Acute MI	Surgical removal	Recovery	4
Rao et al., 1990^56^	71	Female	NR	Constitutional symptoms mostly Dressler’s syndrome following silent MI, masked by RBBB.	Sinus rhythm with RBBB then AF	None	Acute silent MI	Surgical removal	Recovery	4
Zaikokuji et al., 2018^57^	68	Female	Bipolar disorders	Gastric ulcer (upper abdominal discomfort)	ST-segment depression and T wave inversion V3-V6	None	Takotsubo cardiomyopathy	Surgical removal	Recovery (discharged on day 15 postoperative)	4
Ho et al., 2008^58^	29	Male	Cocaine use	Embolic stroke	NR	Brain	MI (substance abuse)	Surgical removal	Recovery	4

**Table 2:** Thrombus characteristics. LV, left ventricle; EF, ejection fraction; NR, not reported; LVOT, left ventricle outflow tract.

**Author/ year**	**LV thrombus Site**	**Thrombus dimensions by echocardiography (Cm)/ (pathology specimen)**	**Other significant echocardiographic findings**
Kumar et al., 2016^9^	Apical and anterior mitral leaflet	3.8* 1.9/ pathology (4*2*1)	EF 50%; hypokinetic apex and apical segment.
Grewal et al., 2021^10^	Apical	1.9*1	Normal systolic function, no segmental wall motion abnormality
Garg et al., 2021^11^	Apical, apical anterior, lateral, and inferior walls.	Largest was 3*3	Moderately reduced LV function
Cousin et al., 2014^12^	Apical	3.3*2.5	EF 5- 10% and dilated LV with global hypokinesia
	Apex, septum, and anterior wall	4.2* 3.5	EF 15- 20%, global hypokinesia, apical akinesia, and dilated right ventricle.
	Apical	2.5* 1.7	Moderate global hypokinesia, severe hypokinesia inferiorly, and inferoseptally.
Kanazawa et al., 2016^13^	Apical	NR	EF 40%, akinesia from the anteroseptal wall to the apex.
Allende et al., 2011^14^	Mid-anterior wall	2.5*1.4	On the first day, normal systolic function and mild left atrial enlargement. On the second day, EF was 52% with hypokinesia of the apical segments.
Lutz et al., 2007^15^	Apical	1.3*1.7 (1*0.9*1.7 after excision)	Normal LV systolic function
Nili et al., 1988^16^	interventricular septum	4*3	EF 26%, dilated LV, akinetic septum, and dyskinetic anterior wall.
	Apical	2*2	EF 28%,
	NR	NR (two pedunculated thrombi)	NR
	NR	1.5*2	NR
Kharwar et al., 2014^17^	Interventricular septum	2.5*2	EF 32%, global hypokinesia, and dilated four chambers.
Ito et al., 2022^18^	Ventricular free wall.	NR	Ef 25% and anterior wall akinesia
Singal et al., 2021^19^	Apical	3.4*1.6	Biventricular systolic dysfunction (EF of 20%), global hypokinesia, and severe central MR
Tanaka et al., 2014^20^	Apical	3*3	EF 10%
Jeganathan et al., 2011^21^	Apical	3*1	Normal
Janula et al., 2021^22^	Apical	NR	EF 35% with akinetic septum, anteroseptal, and anterior walls.
Marchini et al., 2009^23^	Interventricular septum	4.6* 1.2	EF 41%, an akinetic distal portion of the septum, anterior wall, and the whole apex.
Mukai et al., 1991^24^	lateral wall (near the apex)	1*1 (1.2*1*0.7)	EF 44%, global hypokinesia, akinetic apex, and dilated LV
Park et al., 1986^25^	Apical	7*3 (7*4.5*2.7)	Slightly enlarged LV cavity, akinetic apex, and distal septum
Bakhtiari et al., 2012^26^	Posterior wall and anterolateral wall (mid-segment)	Posterior wall: 2.5*1.8 (2.5*2 after excision). Anterolateral wall 2.8*2.2 (3*4 after excision)	Mild LV dilatation with LVEF 35-40%, akinetic basal inferior, and hypokinetic lateral wall (basal and mid segments)
Chen et al., 1981^27^	Apical	NR	dilated LV with global hypokinesia.
Rester et al., 2001^28^	Apical	2.1*2.5	LVEF decreased from 40% (anteroseptal and inferior wall hypokinesia, LA enlargement, mild MR, and mild TR) to 25%.
Azari et al., 2021^29^	Apical	1.7*1.9	LVEF 40%
Chen et al., 2008^30^		NR	NR
Kumar et al., 2022^31^	Apical	3.4*1.1	Normal LVEF
Eren et al., 2013^32^	Apical	1.8*0.8	Normal (LVEF 67%)
Daley et al., 1987^33^	Posterior wall	2	Normal
John et al., 1991^34^	Apical	1*2.5	Normal
	Apical	1*1.5	Anteroapical akinesia
Lewin et al., 1980^35^	Anterolateral	NR	Anterior wall aneurysm.
Shetty et al., 2011^36^	posterolateral papillary muscle	NR	Normal
Vaganos et al., 1989^37^	Apical	3.6*2.9*1.1 (after excision)	Normal
Lew et al., 1983^38^	Apical	1.5	LV dilatation and septal hypokinesia.
Chamsi-Pasha et al., 2009^39^	Apical (apical septal)	1.5*2.7	LVEF 35%, global hypokinesia, and mild mitral regurgitation.
Early et al., 2001^40^	Apical	2*2	LVEF 25% and akinetic apex
Wohlfarter et al., 1991^41^	Septum	3.5*2	NR
Palazzuoli et al., 1994^42^	Apical	1.5 at the longest diameter	NR
Jeon et al., 2012^43^	Apical	4.3*4.2	Global systolic dysfunction with LVEF 19% and dilated LV (6.5 cm).
Chirillo et al., 1996^44^	Apical	10 at the longest dimension.	NR
DeWitt et al., 1988^45^	Apical	2*2.5	Normal
Hwang et al., 1985^46^	Apical	4 to 5	LVEF 26% and anterior apical aneurysm.
Çil et al., 2013^47^	Apical (apical septal)	4*1.1	Left ventricle dysfunction (LVEF 26%) and apical aneurysm
Seitz et al., 2012^48^	Apical (apical inferior)	2.8*1.6 (3*1.5)	Left ventricle dysfunction with apical to mid-anterior hypokinesia.
Manasrah et al., 2022^49^	Apical	Apical thrombus measuring 1.8*1.2 (2.3*2.1 by TEE at the anterolateral wall) and 1*0.5 (Apex)	Normal LV function with no segmental wall motion
Jeganathan and Ralph‐Edwards, 2011^50^	Apical	3*1	Normal
Erkal et al., 2017^51^	Septum	1.3*1.1	LVEF 65%
Maruri-Sánchez et al., 2019^52^	Septum	1.6*1.7	Normal (LVEF 60% with no regional wall motion)
Muller et al., 1996^53^	Apical	4*2	Normal LV function
Lin et al., 2004^54^	Between the septum and inferior wall close to the LVOT	1. 3*2 (3*2*2)	Normal
		2. 1*1 (1*1*0.5)	
Kuroki and Murakami, 2012^55^	Apical	1. 1.5*1.4*1.3	Apical and anteroseptal wall motion and mild mitral regurgitation
		2. 0.3*0.2*0.1	
Rao et al., 1990^56^	Apical	NR	On day 2: mid and apical anterior akinesia
Zaikokuji et al., 2018^57^	Apical	NR	Highly mobile, pedunculated mass arising from the left ventricular apex and protruding into the mitral orifice
Ho et al., 2008^58^	Apical	1.9*1.8 (decreased to 1.8*1.1 on the 5^th^ day but became more mobile)	LVEF 42.1%; akinetic apical and mid segments
	Apical	2.5*1.5	LVEF 45%; apical anterior dyskinesia; apical septal, inferior, and lateral hypokinesia.
